# Oxidative and pro-inflammatory impact of regular and denicotinized cigarettes on blood brain barrier endothelial cells: is smoking reduced or nicotine-free products really safe?

**DOI:** 10.1186/1471-2202-15-51

**Published:** 2014-04-23

**Authors:** Pooja Naik, Neel Fofaria, Shikha Prasad, Ravi K Sajja, Babette Weksler, Pierre-Olivier Couraud, Ignacio A Romero, Luca Cucullo

**Affiliations:** 1 Department of Pharmaceutical Sciences, Texas Tech University Health Sciences Center, School of Pharmacy, 1300 S. Coulter Street, Amarillo TX 79106, USA; 2 Center for Blood Brain Barrier Research, Texas Tech University Health Sciences Center, Amarillo, TX 79106, USA; 3 Department of Biomedical Sciences, Texas Tech University Health Sciences Center, Amarillo, TX 79106, USA; 4 Weill Cornell Medical College, New York, NY, USA; 5 Inserm, U1016, Institut Cochin, Paris, France; 6 CNRS, UMR8104, Paris, France; 7 Université Paris Descartes, Sorbonne Paris Cité, Paris, France; 8 Department of Life, Health and Chemical Sciences, Open University, Milton Keynes, UK

**Keywords:** Tobacco, *In vitro*, Smoking, Oxidative stress, Blood–brain barrier, Inflammation, Nicotine, Permeability, Nicotine Free, Ultralow nicotine, alternative

## Abstract

**Background:**

Both active and passive tobacco smoke (TS) potentially impair the vascular endothelial function in a causative and dose-dependent manner, largely related to the content of reactive oxygen species (ROS), nicotine, and pro-inflammatory activity. Together these factors can compromise the restrictive properties of the blood–brain barrier (BBB) and trigger the pathogenesis/progression of several neurological disorders including silent cerebral infarction, stroke, multiple sclerosis and Alzheimer’s disease. Based on these premises, we analyzed and assessed the toxic impact of smoke extract from a range of tobacco products (with varying levels of nicotine) on brain microvascular endothelial cell line (hCMEC/D3), a well characterized human BBB model.

**Results:**

Initial profiling of TS showed a significant release of reactive oxygen (ROS) and reactive nitrogen species (RNS) in full flavor, nicotine-free (NF, “reduced-exposure” brand) and ultralow nicotine products. This release correlated with increased oxidative cell damage. In parallel, membrane expression of endothelial tight junction proteins ZO-1 and occludin were significantly down-regulated suggesting the impairment of barrier function. Expression of VE-cadherin and claudin-5 were also increased by the ultralow or nicotine free tobacco smoke extract. TS extract from these cigarettes also induced an inflammatory response in BBB ECs as demonstrated by increased IL-6 and MMP-2 levels and up-regulation of vascular adhesion molecules, such as VCAM-1 and PECAM-1.

**Conclusions:**

In summary, our results indicate that NF and ultralow nicotine cigarettes are potentially more harmful to the BBB endothelium than regular tobacco products. In addition, this study demonstrates that the TS-induced toxicity at BBB ECs is strongly correlated to the TAR and NO levels in the cigarettes rather than the nicotine content.

## Background

Tobacco smoke (TS) is a major public health hazard, accounting for more than 5.4 million premature deaths worldwide and over 440,000 deaths each year in the United States alone [1]. In addition to the onset of various forms of cancer [2], smoking has been associated with the pathogenesis and/or progression of a number of major neurological disorders. These include, but are not limited to, silent cerebral infarction (SCI) [3], stroke [4] due to the pro-coagulant and atherogenic effects of smoking [5,6] and cerebral aneurysms [7]. There is also a strong correlation between smoking and an increased risk for multiple sclerosis [8,9], Alzheimer’s disease, small vessel ischemic disease (SVID) and neurodevelopmental damage during pregnancy [10]. Although it is possible to explain some of the neuropathological effects of TS with nicotine specific pathways [11], the precise harmful mechanisms activated by tobacco smoke remain unclear. Thus the neuropathology of cigarette smoking and underlying pathogenic pathways remain largely unknown, although TS-dependent impairment of blood–brain barrier (BBB) function is certainly a critical prodromal factor.

A burgeoning yet incomplete body of evidence suggests that cerebrovascular inflammation and impairment of endothelial physiology are primarily responsible for a large number of neurological disorders associated with BBB dysfunction [12]. This provides a solid link to TS-dependent impairment of BBB function whereas cigarette smoke extracts have been shown to act as a powerful activator of immune/inflammatory response pathways altering the integrity/function of the BBB [13,14].

Mainstream TS contains over 4000 chemical compounds including a harmful cloud of free radicals and other reactive oxygen (ROS) and nitrogen species (RNS) contained in both the gaseous phase and the tar [15]. At the vascular level free radicals can lead to oxidative damage of endothelial cells [16] involving DNA strand breakage and inflammation [17-19]. Active and passive tobacco smoking can spawn these highly reactive oxygen species (hydrogen peroxide, epoxides, nitric oxide (NO), nitrogen dioxide, peroxynitrite (ONOO) [20]) beyond the levels which the human body can eliminate effectively. In fact, several studies have shown that: 1) chronic smokers suffer from antioxidant shortage caused by increased anti-oxidative mobilization in response to systemic oxidative stress evoked by ROS-enriched TS [21,22]; 2) antioxidant supplementation reduces the oxidation and inflammation induced by TS in animals and cells [14,23]; 3) TS contributes to a pro-atherosclerotic environment by triggering a complex pro-inflammatory response and mediates the recruitment of leukocytes [24] through cytokine signaling.

The tobacco industry has developed “reduced exposure” and “light” products containing lower levels of nicotine, nitrosamines or other chemicals deemed to be potentially toxic. However, experimental and clinical data supporting the claim that these products reduce the health hazard of tobacco smoking are lacking. To date, only a handful of studies have investigated the effect of TS on BBB function and integrity, thus limiting our understanding of mechanisms involved in TS-related toxicity at BBB and associated risks for neuropathological disorders.

Therefore, in our study we investigated the effects of various tobacco products (including ultralow nicotine and tobacco-free cigarettes) on BBB endothelium *in vitro,* using a well characterized human BBB endothelial cell line (hCMEC/D3; [25,26]. Data from this study indicates that smoking-related dysfunction of BBB endothelial physiology (e.g., increased oxidative stress, impaired tight junction expression/distribution, etc.) positively correlate with the total content of tar of various tobacco products and associated oxidative stress (ROS and NO output) rather than nicotine content.

## Results

### Exposure to nicotine concentrations equivalent to that observed in plasma in chronic human smoker does not affect endothelial cell viability

**Figure 1:**
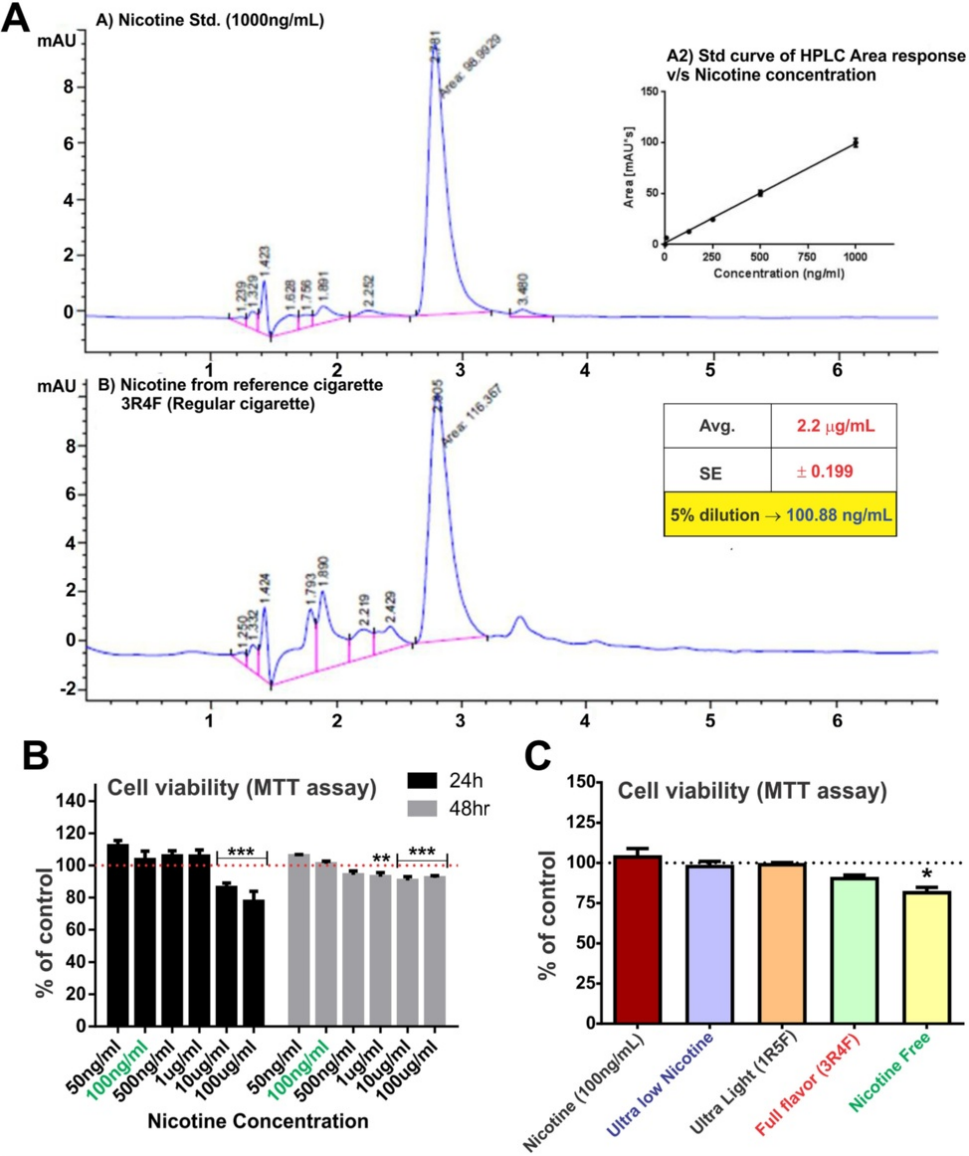
**HPLC and viability studies to select the CSE concentration for the study. A)** HPLC analysis to determine nicotine concentration in CSEshowed that 5% CSE had nicotine concentration comparable to the physiological concentration in a chronic smoker (100 ng/ml) n = 10. **(B)** Cell viability studies following increasing concentration of nicotine at 24 and 48 h and **(C)** 5% diluted CSE from ultralow, 1R5F (equivalent to ultralight cigarettes), 3R4F (equivalent to full flavor cigarettes), ultralow nicotine and tobacco free (nicotine free - NF) using MTT assay. Note that 5% CSE from all tested brand but NF did not cause a statistically significant decrease in cell viability. n = 3 individual experiments.

HPLC studies were performed to determine the dilution factor for freshly prepared 3R4F cigarette-derived CSE stock solution necessary to achieve CSE exposure yielding 100 ng/ml of nicotine (Figure 1A). This nicotine concentration was chosen to model the plasma levels seen in human smokers [27-29]. 3R4F cigarette was used as a reference to calculate the dilution factor for the CSE stock which was then uniformly applied to all the test cigarettes. As shown in Figure 1B, 100 ng/ml of nicotine did not affect the cell viability at 24 and 48 h exposure. Cytotoxic effects of nicotine exposure were observed at higher concentrations (10 and 100 μg/ml/24 h; 1, 10, 100 μg/ml/48 h). Note also that 24 h exposure to 5% diluted CSE from test cigarettes did not affect endothelial viability with the exception of NF-derived extracts (see Figure 1C). A small yet significant decrease in cell viability was observed in response to NF-derived CSE exposure, as compared to controls (CSE-free PBS or 100 ng/ml nicotine treatment).

### Nitrate and nitrite levels in CSE correlates with corresponding cigarette’s tar and nicotine content

**Figure 2:**
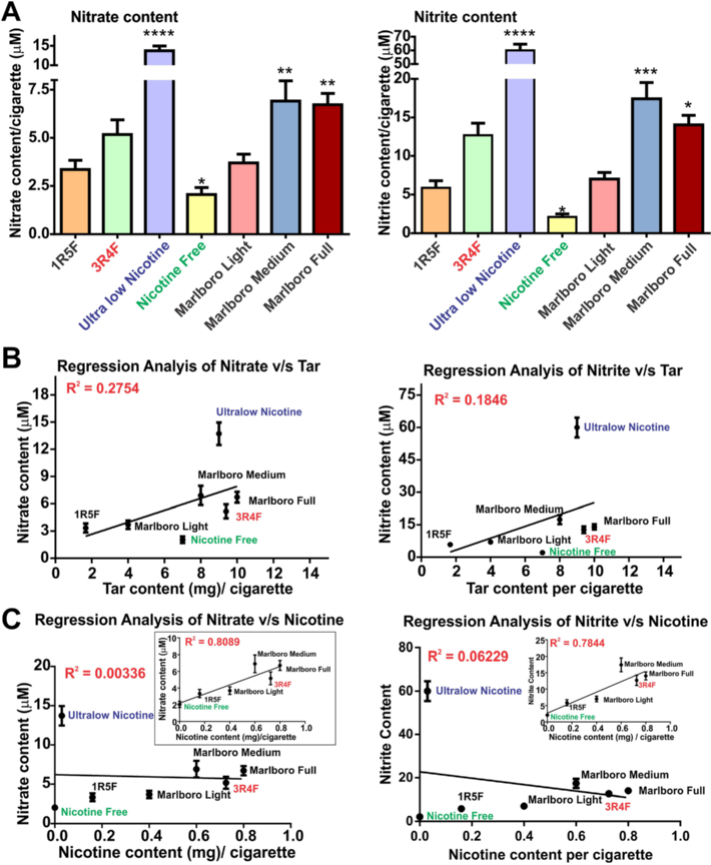
**Nitrate/Nitrite content profiling of CSE from tested tobacco products. (A)** Nitrate/nitrite content increased proportionally to the amount of tar in the cigarettes where statistically significant higher nitrate was found in ultralow nicotine than ultralight cigarette (P < 0.001, n = 10 biological replicates). Nicotine free cigarettes which were non-tobacco based did not have significant nitrate/nitrite content. Regression analysis of Nitrate/Nitrite correlates with tar **(B)** but not nicotine content unless ultralow nicotine cigarettes are removed from the analytical pool **(C)**.

A generation of highly carcinogenic tobacco-specific nitrosamines (TSNA) [30] has been suggested to arise from the reaction of amines with nitrite derived from nitrate in the tobacco [31]. We measured nitrate, NO^3−^/nitrite andNO^2−^ content of CSE derived from 1R5F (ultralight), 3R4F (full flavor), Ultralow nicotine and NF (a non-tobacco based product) cigarettes (Figure 2A). In addition, commercially available Marlboro light, medium and full cigarettes were also analyzed for comparison. NO^3−^/NO^2−^ content in CSE from ultralow nicotine cigarettes was significantly higher than any other brand tested including 3R4F (p < 0.0001, compared to light cigarettes), as observed with Marlboro full or medium cigarettes (p < 0.01, compared to light cigarettes, Figure 2A). In contrast, NO^3−^/NO^2−^ content of NF cigarette was significantly lower (p < 0.05) when compared to “light” cigarettes (Figure 2A). A positive correlation between NO^3−^/NO^2−^ content of CSE and tar for the corresponding cigarette brands was also observed as demonstrated by the regression analysis shown in Figure 2B. Importantly, the ultralow nicotine brand stands out in terms of NO^3−^/NO^2−^ content when compared to other brands containing a similar amount of tar. NO^3−^/NO^2−^ positively correlate with the nicotine content of corresponding cigarette types with an exception of the ultralow nicotine brand (Figure 2C and insets). Results from the regression analyses (tar and nicotine versus NO^3−^/NO^2−^) suggests that an alteration of the tobacco product to reduce the nicotine content, such as in ultralow nicotine and NF brands, could be responsible for the higher output of NO^3−^/NO^2−^ during cigarette combustion.

### Release of hydrogen peroxide (H_2_O_2_) in CSE increases with the tar content of cigarettes and leads to progressive oxidative damage in BBB ECs

**Figure 3:**
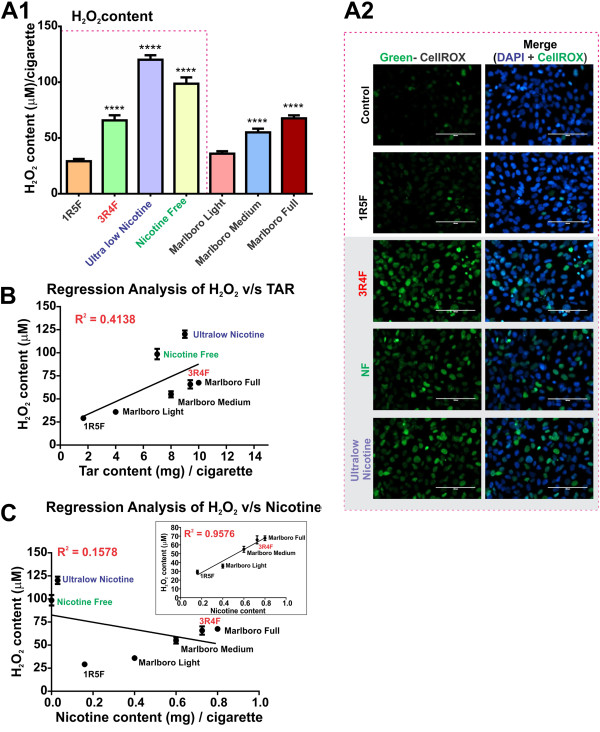
**Hydrogen Peroxide content profiling of CSE from tested tobacco products. (A1)** H_2_O_2_ content increased proportionally to the amount of tar in the cigarettes where statistically significant higher H_2_O_2_ was found in full flavor, ultralow nicotine and nicotine free cigarettes than light cigarette (P < 0.001), (n = 10 CSE preparations). **(A2)** Immunofluorescence analysis of oxidative stress in ECs (HCMEC/D3 cell line) caused by CSE exposure from 1R5F, 3R4F, ultralow nicotine and NF cigarettes versus controls: Note that most significant oxidative responses were observed in EC cultures exposed to CSE treatment (24 h) derived from 3R4F, ultralow nicotine and NF cigarette (n = 3 biological replicates). Regression analysis of H_2_O_2_ correlates with tar **(B)** but not nicotine content unless ultralow nicotine and NF cigarettes are removed from the analytical pool **(C)**.

TS is a major exogenous source of free radicals contained in both gaseous phase and tar, which can spawn sustained high levels of ROS (e.g., H_2_O_2_) that may directly affect the BBB integrity. Thus, we determined the amount of H_2_O_2_ release in the CSE and assessed its oxidative effect on BBB endothelial cell cultures. As shown in Figure 3A1, the highest levels of H_2_O_2_ were found in CSE from ultralow nicotine and NF (tobacco-free) cigarettes when compared to light products (p < 0.0001). H_2_O_2_ content in CSE from 3R4F (full flavor) and Marlboro medium/full cigarettes, although statistically higher that light cigarettes, was considerably less compared with ultralow nicotine and NF products. This was in accordance with measurements of cellular oxidative stress (CellROX® Green Reagent, Figure 3A2) and revealed the highest level of cellular oxidation in endothelial cultures chronically exposed (24 h) to Ultralow nicotine and NF smoke extracts. Interestingly, 3R4F cigarettes released lower amounts of H_2_O_2_ than Ultralow nicotine and NF cigarettes which also demonstrated a comparable oxidative stress potential in endothelial cells. Furthermore, regression analysis of H_2_O_2_ content revealed a direct relationship between tar content of the corresponding cigarette brand (Figure 3B) and H_2_O_2_. A positive correlation between H_2_O_2_ and nicotine content was also observed but only with the exclusion of NF and ultralow cigarettes from the analytical pool (Figure 3C).

### Exposure to CSE from 3R4F, NF and ultralow nicotine cigarettes negatively impacts ZO-1 and occludin expression/distribution as well as BBB integrity

**Figure 4:**
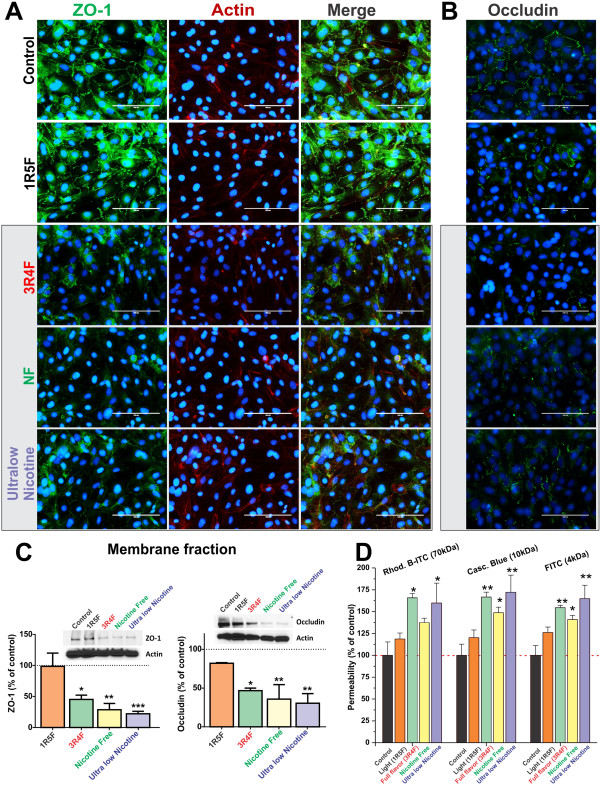
**Effect of CSE exposure (24 h) on endothelial expression and distribution of ZO-1, Occludin and actin filaments. (A)** Down-regulation of ZO-1 expression and disruption and cell-cell junctions were progressively more significant following exposure to 3R4F, NF and ultralow nicotine CSEs. Down-regulation and disruption at cell-cell contacts of occludin were also observed **(B)**. Immunofluorescence analyses were confirmed by WB of corresponding membrane fractions **(C)**. Loss of BBB integrity was further assessed by permeability measurements to dextran molecules ranging from 3 to 70 kDa **(D)** n = 3 biological replicates, *p < 0.05, **p < 0.01, ***p < 0.001 compared to controls.

Immunofluorescence analysis of BBB endothelial confluent monolayers revealed a significant loss of ZO-1 at cell-cell junctions following exposure to CSE from 3R4F, Ultralow and NF cigarettes and to a lesser extent in 1R5F treated cultures (compared to controls, Figure 4A). Results were further confirmed by western blot (WB) analysis of the corresponding membrane fractions (Figure 4C, left panel) in which exposure to ultralow nicotine CSE caused the most significant reduction of ZO-1 expression at the membrane level (p < 0.0005, Figure 4C). However, actin distribution and expression were not altered by CSE exposure when compared to controls. Parallel immunofluorescence analyses also revealed a similar down-regulation and altered pattern of distribution at cell-cell junction for occludin (Figure 4B). Results were also confirmed by WB analysis of occludin expression in the corresponding membrane fractions (Figure 4C, right panel). Expression levels of occludin were severely impaired in endothelial cultures exposed to CSE from 3R4F cigarette. The effect was even more significant in culture exposed to NF and ultralow nicotine smoke extracts (see Figures 4B & C). Note also that a modest (not statistically significant) alteration in the distribution and expression level of occludin was observed in cell cultures treated with 1R5F-derived CSE when compared to controls. As expected, alteration in TJ expression/distribution impacted BBB integrity as demonstrated by permeability measurements to dextran molecules (see Figure 4D). Increased permeability to 70 kDa dextran was noted in Transwells exposed to TS extracts from NF, ultralow and 3R4F although results were deemed significant (p < 0.05) only for the last two conditions. On the other hand, permeability to lower molecular weight dextrans (10 and 4 kDa) was significantly increased for all the three conditions mentioned above.

**Figure 5:**
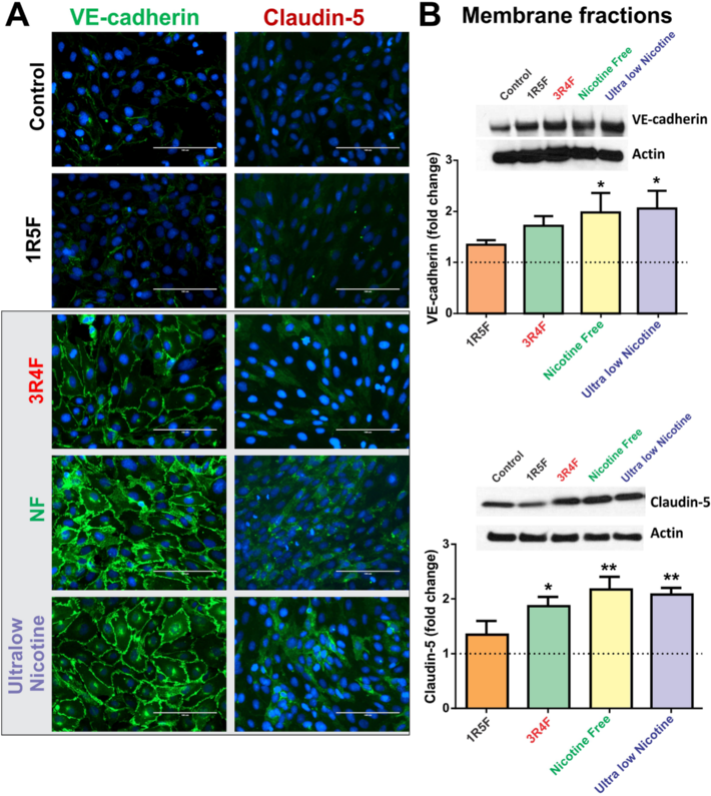
**Effect of CSE exposure (24 h) on endothelial expression and distribution of VE-cadherin and Claudin-5. (A)** Immunofluorescence analysis of BBB endothelial cultures revealed a significant up-regulation of VE-cadherin (at cell-cell junctions) and claudin-5 expression following exposure to 3R4F, NF and ultralow nicotine CSEs. **(B)** Immunofluorescence analyses were confirmed by WB of corresponding membrane fractions. n = 3 biological replicates, *p < 0.05, **p < 0.01 compared to controls.

Further, immunofluorescence analysis revealed a marked up-regulation of VE-cadherin at cell-cell contacts in response to CSE exposure from 3R4F, NF and ultralow nicotine (Figure 5A). Results were confirmed by parallel WB analysis of corresponding membrane fractions (Figure 5B) demonstrating a statistically significant (p < 0.05) increase in VE-cadherin expression following exposure to NF and ultralow nicotine derived CSE, compared to controls and cultures exposed to 1R5F. A noticeable (although not statistically significant) increase in VE-cadherin expression was also observed in endothelial cultures treated with 3R4F-derived CSE. Similarly, claudin-5 expression was up-regulated by exposure to CSE derived from 3R4F, ultralow nicotine and NF cigarettes, as demonstrated by immunofluorescence and WB analyses of corresponding membrane fractions (Figure 5A & B). Interestingly, the patterns/level of expression of these junction proteins remarkably reflects the oxidative stress/H_2_O_2_ content of the CSE extracts of the respective brands (as shown in Figure 3A1 & A2).

### Exposure to CSE from 3R4F, NF and ultralow nicotine cigarettes promotes the pro-inflammatory activation of BBB endothelial cells

**Figure 6:**
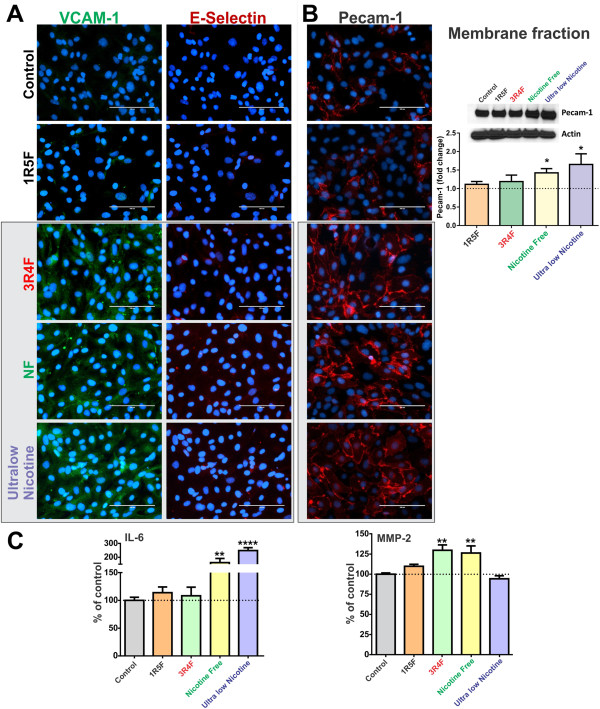
**Immunofluorescence analysis of BBB endothelial expression of VCAM-1 and E-selectin (A) and PECAM1 (B), following exposure to CSEs from 1R5F, 3R4F, NF and ultralow nicotine cigarettes.** Immunofluorescence analysis of PECAM1 was confirmed by WB of corresponding membrane fractions. **(C)** Release of proinflammatory cytokines IL-6 was up-regulated in endothelial cultures exposed to NF and ultralow nicotine CSE while MMP-2 levels were increased by CSE from 3R4F and NF but not ultralow nicotine. n = 3 biological replicates, *p < 0.05, **p < 0.01, ****p < 0.0001 compared to controls.

As shown in Figure 6A, endothelial cell expression of vascular endothelial adhesion molecule-1 (VCAM-1) was up-regulated following exposure to 3R4F, NF and ultralow nicotine CSEs, as compared to controls and 1R5F cigarette treated cultures. However, no expression changes were observed with respect to E-selectin. In addition, immunofluorescence analysis indicated an up-regulation of endothelial Platelet Endothelial Cell Adhesion Molecule-1 (PECAM1) expression following exposure to 3R4F, NF and ultralow nicotine cigarettes (Figure 6B). These results were further supported by WB analysis of the corresponding membrane fractions (Figure 6B).

Importantly, analysis of the culture conditioned media by ELISA revealed a significant increase of interleukin-6 (IL-6) release from the endothelial cells exposed to either NF (p < 0.01) or ultralow nicotine cigarette extracts (p < 0.005), compared to controls (Figure 6C). A modest, yet significant increase in the release of matrix metalloproteinase-2 (MMP-2) was also observed in cultures treated with either 3R4F or NF smoke extracts, but not ultralow nicotine (Figure 6C). However, MMP-9, IL-1β and TNF-α levels in the conditioned media from all treatment conditions were below the reading sensitivity (data not shown).

## Discussion

ROS, despite being essential for biological systems [32] have the potential to cause extensive oxidative damage to cells and tissues if their levels become excessive [33,34]. At the vascular level ROS can cause oxidative damage of endothelial cells [16] including DNA strand breakage and inflammation [17]. In addition to ROS, nicotine can equally elicit oxidative stress and tissue injury [35,36] and has been shown to exacerbate brain edema following focal ischemia [37,38]. Oxidants in the gaseous phase of cigarette smoke, including nicotine and various ROS species, ([15,20] can pass through the lung alveolar wall and raise systemic oxidative stress [39]. This can lead to oxidative damage to cells and tissues, including the brain vascular system and the BBB, over a period of sustained exposure to TS (e.g., chronic smokers) and facilitate the pathogenesis and progression of neurological disorders [40-42]. Thus, existing evidence strongly suggests a role for TS-dependent oxidative and inflammatory stress in the development of CNS pathologies. In fact, the cerebrovascular endothelium is highly vulnerable to oxidative stress resulting in loss of BBB function and integrity via altered expression and distribution of intercellular TJ complexes [43,44].

In this study we assessed and compared the effects of various tobacco products on human BBB endothelial cells in relation to their corresponding oxidative potential. Specifically, several studies have demonstrated that cigarette smoke contains high concentrations of NO which may directly affect the integrity of the BBB. For this purpose we measured ROS as well as NO^3−^/NO^2−^ content (Figures 2 & 3) of tobacco smoke from 1R5F (ultralight), 3R4F (full flavor), NF (tobacco free) and ultralow nicotine cigarettes. The NO^3−^/NO^2−^ analysis revealed a direct correlation with the content of tar of the respective cigarettes. However, this did not hold true for NF products, whose tar content (comparable to medium strength cigarettes) produced the least amount of nitrate and nitrite. When we compared the NO^3−^/NO^2−^ output with corresponding nicotine content, a significant correlation was not found, unless ultralow nicotine brand were removed from the pool (Figure 2C - insets). Together these results suggest that NO^3−^/NO^2−^ is relatively independent of nicotine content while holding a strong correlation with that of tar.

Tar being a byproduct derived from combustion of tobacco or analogous products, alteration of tobacco (e.g., ultralow nicotine products) or replacement with alternative products (NF cigarette) to reduce nicotine content in a bid to decrease addiction potential, may result in an unwanted increase of nitrate/nitrite output, and risk for health hazard. In fact, tobacco nitrate levels have been previously reported to correlate with the formation of non-specific volatile nitrosamines (e.g., N-nitrosodimethylamine, N-nitroso-diethylamine, N-nitrosoethylmethyl- amine, etc.), and non-volatile Tobacco-Specific Nitrosamines (TSNAs) such as 4-(methylnitrosamino)-1-(3-pyridyl)-1-butanone (NNK, nicotine-derived nitrosamine ketone) which have been associated with carcinogenicity of tobacco smoke [30,31].

Interestingly, H_2_O_2_ content measured in ultralow nicotine and tobacco free (NF) cigarettes, considered “reduced-exposure” products, was significantly higher than any other brand including medium and full flavor (see Figure 3). Regression analysis of H_2_O_2_ also revealed a strong correlation with the tar content of the respective cigarettes but not with that of nicotine unless both products were to be removed from the pool. These results strongly correlate with the increased oxidative stress generated in BBB endothelial cultures (see Figure 3A2) and revealing that the highest level of oxidation is in endothelial cells that are exposed to ultralow and NF cigarette smoke extracts. Interestingly, the oxidative stress potential of 3R4F cigarettes was comparable to that of ultralow and NF cigarettes despite releasing lower amounts of H_2_O_2_. This can be attributed to the higher content of nicotine in 3R4F cigarettes since nicotine equally contributes to oxidative stress (Das et al. 2012). Taken together, these results suggest that alteration and/or substitution of tobacco with alternative products in order to reduce nicotine content was responsible for the increased H_2_O_2_ output measured in these “denicotinized” cigarette products.

Previous reports by Hossain and co-workers [14] have shown a dose dependent loss of BBB integrity directly correlating to TS-derived oxidative stress. Furthermore, loss of BBB function and integrity caused by TS exposure was prevented or at least reduced by antioxidant vitamins. These findings by others clearly support our results which outlined a strong correlation between the impairment of tight junction protein expression/distribution and BBB integrity with the oxidative stress generated by the TS extracts. As clearly shown in the results (see Figure 4) BBB endothelial ZO-1 expression and distribution is completely deregulated upon exposure to TS extract from 3R4F, NF and Ultralow Nicotine cigarettes. This is also reflected in the increased BBB permeability to dextran paracellular markers observed under the same conditions.

ZO-1 is a cytoplasmic accessory protein which plays a crucial role in BBB integrity by connecting transmembrane proteins (such as occludin, claudins and JAM) to cytoskeletal proteins and is actively involved in signal transduction and transcriptional modulation [45,46]. Interestingly, the effect of CSE on ZO-1 expression/distribution reflects the overall oxidative potential of the corresponding cigarettes (see Figure 3), thus suggesting a correlation between TS-dependent oxidative potential and dysregulation of TJs and BBB integrity. ZO-1 TJ protein closely associates with the actin cytoskeletal network. When we observed the actin structure with respect to ZO-1, it appeared intact. In addition, membrane expression of occludin was significantly down-regulated as evidenced by the WB analysis of the corresponding membrane fractions. Similar to ZO-1, membrane distribution of occludin was also altered deteriorating from a homogenous pattern at cell-cell junctions in controls to a patchy distribution in cultures exposed to 3RF4, NF and ultralow nicotine cigarettes. This can be a reflection of the parallel loss of ZO-1 which provides a positioning system and anchoring scaffold for the transmembrane TJ proteins.

In contrast to ZO-1 and occludin, the expression of VE-cadherin and claudin-5 was proportionally increased with respect to the oxidative potential of the corresponding CSE treatment. In fact, as shown in Figure 5, VE-cadherin membrane expression was progressively up-regulated by exposure to 3RF4, NF and ultralow nicotine cigarettes, although statistical significance was proven only for the last two cigarette products. In parallel, claudin-5 membrane expression was similarly up-regulated (see Figure 5B). This is in agreement with emerging evidences suggesting that VE-cadherin controls claudin-5 expression by preventing the nuclear accumulation of FoxO1 and beta-catenin which repress the claudin-5 promoter [47] thus reducing its expression. Although, these results were surprising, they actually seem to be in agreement with the above mentioned observations. In fact, recent *in vitro* studies have shown a direct positive correlation between VE-cadherin expression and oxidative stress [48] suggesting this being part of a cytoprotective response mechanism. In fact, VE-cadherin acts as a master regulator of various endothelial functions including modulation of cell-cell adhesion, angiogenesis, and vascular permeability to leukocytes in response to VCAM-1 activation [49], whose expression level was also increased (see Figure 6). Note also that an up-regulation of claudin-5 (in this case mediated by VE-cadherin) does not necessarily translate into an improved BBB integrity. Although this is true from a biological standpoint under normal circumstances we have to take into consideration that the mere expression of TJ proteins is not sufficient as a standalone determinant for BBB integrity. Other important factors play a significant role here such as the link between TJ proteins with the cytoskeleton. An important interaction mediated by first order regulatory proteins such as ZO-1 is of critical importance for the positioning and interaction of TJ proteins with their homologues on adjacent endothelial cells. Moreover, although claudin-5 was up-regulated (see Figure 5) the pattern of expression presented as an homogenous distribution throughout the cells and lacked a demarcated membrane localization which does not suggest improvements of cell-cell adhesion. This hypothesis well copes with the evident loss of barrier functions outlined by the increased permeability to dextran markers.

In addition, a similar increase in PECAM-1 expression was observed as well as an increased endothelial release of IL-6 and MMP-2 (see Figure 6). Regarding MMP-2, previous reports by others have shown how ROS regulate the activity of vascular matrix metalloproteinases *in vitro* including MMP-2 and MMP-9 [50] which have an implication in atherosclerotic plaque stability. Expression and activation of MMP-2 has been demonstrated as a key event in oxidative stress injury to heart [51] and hyperglycaemia promoted BBB dysfunction [52]. Together these results strengthen the link between tobacco smoke, it’s corresponding oxidative and inflammatory stress, and potential risk for BBB dysfunction. Although outside the scope of the present work, more studies will be necessary to dissect the molecular mechanisms involved in the generation of cellular oxidative stress at the brain microvascular endothelium by CSE and its impact on BBB function and integrity.

## Conclusion

In summary, this study is one of the first attempts to assess and compare the potential toxic impact of various cigarette products on BBB endothelial cells using whole smoke extracts. We further correlated the oxidative and inflammatory potential of these cigarette products with respect to their tar, nicotine, H_2_O_2_ and nitric oxide content. We also clearly showed that the alteration of tobacco in an attempt to reduce cigarette nicotine content to attenuate addiction can result in an increased toxicity and endothelial inflammatory response. This can ultimately impair the BBB function and increase the risk for the pathogenesis of a number of CNS disorders.

## Methods

### Materials and reagents

The antibodies used in this study were obtained from the following sources: Rabbit anti-ZO-1 (#8193), rabbit anti-claudin-3 (#341700), rabbit anti-VE-cadherin (#D87F2), rabbit anti-VCAM-1 (#12367), mouse PECAM-1 (#89C2) from Cell Signaling Technology (Danvers, MA, USA); mouse anti-E-selectin (#S 9555), β-actin (#A5441) from Sigma-Aldrich (St. Louis, MO, USA); donkey anti-rabbit (#NA934) and sheep anti-mouse (#NA931) HRP-linked secondary antibodies from GE Healthcare (Piscataway, NJ, USA); mouse anti-claudin 5 (#35-2500), goat anti-rabbit (#A11008) and anti-mouse (#A21422) conjugated to Alexa Fluor® 488 and 555 from Invitrogen (Camarillo, CA, USA). Sterile cultureware was obtained from Fisher Scientific (Pittsburgh, PA, USA), while other reagents and chemicals were purchased from Sigma-Aldrich (St. Louis, MO, USA) or Bio-Rad laboratories (Hercules, CA, USA).

### TS preparation

**Figure 7:**
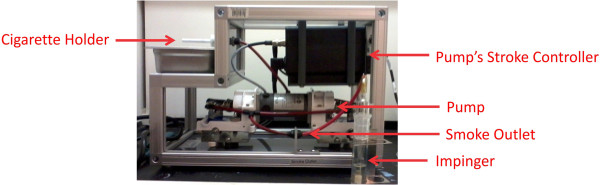
**Smoke Preparation according to ISO/FTC protocol.** Concentrated Smoke solution was prepared from each cigarette using CSM-SCSM Cigarette Smoking Machine according to ISO/FTC determination parameters. These require a puff volume of 35 ml with duration of 2 s at interval of 60s.

**Figure 8:**
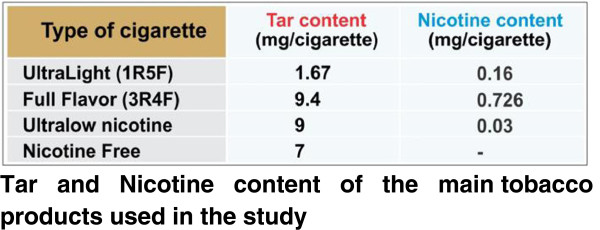
**Tar and Nicotine content of the main tobacco products used in the study.** The following table states the tar and nicotine composition of the various cigarettes used in the study. (^+^ 1R5F and 3R4F are the names of these cigarettes provided by University of Kentucky for research purposes).

Concentrated cigarette smoke extracts (CSE) were prepared according to the Federal Trade Commission (FTC) standard smoking protocol (35 ml draw, 2 second puff duration, 1 puff per 60 seconds), using a Single Cigarette Smoking Machine (SCSM, CH Technologies Inc., Westwood, NJ, USA), as shown in Figure 7. This protocol resulted in approximately 8 puffs per cigarette. Mainstream cigarette smoke was bubbled through an impinger into phosphate buffered saline (PBS) to generate a concentrated CSE stock (100%) solution that was further diluted to desired concentrations and was used immediately for the experiments described below. Four types of cigarettes were used for this study, as shown in Figure 8: a) 1R5F cigarettes equivalent to commercial ultralight brands with 1.67 mg tar and 0.160 mg nicotine per cigarette b) 3R4F cigarettes equivalent to full flavor brands with 9.4 mg tar and 0.726 mg nicotine per cigarette (obtained from University of Kentucky); c) reduced nicotine spectrum cigarettes (obtained from NIH/NIDA) equivalent to ultralow nicotine brands with 0.03 mg nicotine and 9 mg tar per cigarette; and iv) commercially available nicotine-free cigarette from non-tobacco based source with a tar content of 7 mg per cigarette. Commercially available light, medium and full flavor cigarettes from Marlboro were also used for comparative profiling experiments.

### Cell culture

The immortalized hCMEC/D3 cell line was donated by Dr. Couraud (INSERM, Paris). The hCMEC/D3 cells (passage 28–32) were seeded on collagen coated culture flasks (2.5-3 × 10^4^/cm^2^) or glass slides (4 × 10^4^/cm^2^) and maintained at 37°C with 5% CO_2_ exposure in EBM-2 basal medium (Lonza, Walkersville, MD, USA) supplemented with 5% FBS (Atlanta Biologicals, Lawrenceville, GA, USA), chemically defined lipid concentrate (Life Technologies, Carlsbad, CA, USA), growth factors, antibiotic/antimycotic (1:1, Atlanta Biologicals, GA, USA and HEPES (10 mM). Medium was changed every 2 days until the cells reached confluence. Monolayer integrity of hCMEC/D3 cells at confluence was confirmed by phase contrast microscopy and the expression of endothelial cell-specific phenotypic markers at cell-cell junctions, as previously described [26]. For treatment, cell monolayers were exposed to CSE concentration (5-20%) diluted from freshly prepared smoke extracts as described above. Cultures exposed to CSE-free vehicle (PBS) served as controls.

### Cell viability assay

The effects of CSE exposure on cell viability were determined by MTT (3 (4,5-dimethylthiazol-2yl)-2,5-diphenyltetrazolium bromide) assay. Briefly, HCMEC/D3 cells were passaged in a 96-well plate and allowed to attach for a period of 48 h. Following exposure to CSE, cells were incubated with 10 μM MTT for 5 h at 37°C. MTT was removed and DMSO was added to solubilize the formazan crystals for 20 min. Color development corresponding to viable cells was quantitated by measuring absorbance at 520 nm.

### Nitric oxide (NO) content analysis

Cigarette smoke was bubbled through an impinger into PBS using the CSM-SCCM smoking machine as described above. NO content of the different types of cigarettes was determined indirectly through the estimation of nitrate/nitrite content using Griess reagent reaction based NO kit from R&D Systems, according to manufacturer’s guidelines.

### Hydrogen peroxide (H_2_O_2_) content analysis

H_2_O_2_ content in smoke extracts of various types of cigarettes was determined by TBR4100 free radical analyzer with 100 μm HPO sensor. Briefly, aliquots from cigarette preparation were titrated in PBS to obtain a sensor reading which was extrapolated against a hydrogen peroxide standard curve to quantitate the amount per cigarette.

### HPLC analysis of CSE preparation

For sample preparation, CSE obtained from CSM was subjected to liquid/liquid extraction using dichloromethane. Briefly, 500 μl aliquot of CSE was mixed with 5 μl of 1 M NaOH followed by the addition of 2 ml DCM. After centrifugation of the mixture at 1500 g for 10 min, the upper aqueous layer was discarded. The lower organic layer was evaporated under nitrogen gas, and the precipitate was resuspended in mobile phase, filtered and then injected on to the column. Nicotine (Sigma Aldrich, St. Louis, MA, USA #36733) dissolved in mobile phase was used to prepare the standard curve. Isocratic separation was performed on Agilent A1220 HPLC System (Agilent Technologies, Santa Clara, CA, USA) coupled to a UV detector, using Zorbax Rx-C18 column (4.6x150mm, 5 μm) with an inline guard column filter. The mobile phase consisted of 50 mM KH_2_PO_4_ buffer with 10 mM sodium heptane 1-sulfonate, pH adjusted to 3.0 using orthophosphoric acid and methanol (70:30 v/v). The flow rate was set to 1 ml/min with column temperature at 30°C and injection volume was 50 μl. Wavelength corresponding to maximum absorption of nicotine (259 nm) was used.

### ELISA

Following exposure to CSE, the cell culture conditioned media was collected and stored at −20°C until analysis. Levels of pro-inflammatory cytokines such as IL-1b, TNF-alpha, IL-6 and matrix metalloproteinases (MMPs) such as MMP-2 and MMP-9 were measured by Quantikine ELISA kits from R&D systems as per manufacturer’s instructions.

### Immunofluorescence analysis

Cells were grown in two-well chamber slides specifically for these studies. After treatment, cells were fixed with formaldehyde (15mins at 4°C). Following three PBS washes, cells were blocked using 5% goat serum (Sigma-Aldrich, St. Louis, MO, USA) in PBS at room temperature for 50 min and incubated with primary antibodies prepared in 5% GSA overnight at 4°C. After three rinses with PBS, cells were incubated for 1 h at RT with Alexa Fluor® 488 or 555 conjugated goat anti-rabbit or anti-mouse antibodies, respectively (1:1000). Thereafter, cells were rinsed and counterstained with DAPI in Prolonged Gold Anti-fade mounting media (Invitrogen, OR, USA). Slides were cover slipped and left for overnight drying in the dark before examination with EVOS digital inverted fluorescence microscope. Cells stained with secondary antibodies alone were used as negative controls.

### Western blotting

Briefly, cells were lysed in ice-cold Urea-Tris buffer containing Phosphatase and Protease Inhibitors (Roche Diagnostics, Indianapolis, IN, USA), sonicated and centrifuged at 14000 rpm, 4°C for 15 min. Protein concentration was determined by Bradford assay (Bio-Rad laboratories (Hercules, CA, USA # 5000006). Denatured samples containing equal protein (40 μg) were subjected to SDS-PAGE (10% or 4-15% gradient gel) and electrotransferred to PVDF membranes (2 hr transfer at 100 V). Membranes were blocked for 2 h (RT) with 5% non-fat dry milk in Tris buffered saline (TBS) containing 0.1% Tween-20 (TTBS) and subsequently incubated with rabbit (1:1000) or mouse (1:500) primary antibodies. After 4 washes (10 min each) with TTBS, membranes were incubated with anti-rabbit or anti-mouse (1:2000) HRP-conjugated secondary antibodies (2 h, RT) and washed with TTBS. Bands were detected by enhanced chemiluminescence using Amersham ECL™ Prime with ChemiDoc™ XRS system. Membranes were subsequently stripped and probed for β-actin (1:1000) as a loading control. Band densities were analyzed by Quantity One Software.

### Measurement of BBB integrity: dextran permeability

Differential effects of TS exposure on BBB integrity was assessed by measuring paracellular permeability (luminal to abluminal) to labeled dextrans (4-70 kDa) as previously described [53]. After 24 h exposure to TS extracts, a mixture of labeled dextrans in PBS (FITC- 4 kDa, 5 mg/ml; Cascade Blue®- 10 kDa, 5 mg/ml; and Rhod. B-ITC - 70 kDa, 5 mg/ml) was added to the luminal compartment. Abluminal samples (50 μL) were collected over 30 min and replaced with equal volume of fresh media to allow sink conditions. Dextran fluxes were determined by fluorescent measurements using the appropriate excitation and emission wavelengths. Permeability measurements were reported as percentage of controls (the permeability coefficients of controls were as follow: FITC 0.198 ± 0.009 × 10^−3^ cm/min; Cascade Blue® 0.0953 ± 0.007 × 10^−3^ cm/min and Rhod. B-ITC 0.007 ± 0.0005 × 10^−3^ cm/min).

### Statistical analyses

Data from all experiments were expressed as mean ± standard error of mean (S.E.M) and analyzed by one-way ANOVA using GraphPad Prism Software Inc. (La Jolla, CA, USA). *Post hoc* multiple comparisons were performed with Tukey’s test. P values ≤ than 0.05 were considered statistically significant.

## Competing interests

The authors declare that they have no competing interests.

## Authors’ contributions

PN conceived the study and elaborated its design. PN also performed most of the experiments and drafted the manuscript. NF carried out major western blot analysis. SP carried out (with PN) immunofluorescence and permeability studies. RS provided direction to this project and helped draft the manuscript. BW, PC and IR provided the necessary cell line to conduct these studies. They also assisted with data analysis and manuscript editing/revisions. LC supervised the project, the data analysis and provided guidance during manuscript preparation and revisions. All authors have read and approved the final version of the manuscript.

## References

[B1] Smoking-attributable mortality, years of potential life lost, and productivity losses--United States, 2000–2004 MMWR Morb Mortal Wkly Rep 2008 15 1226 1228 19008791

[B2] Hecht SS Cigarette smoking: cancer risks, carcinogens, and mechanisms Langenbecks Arch Surg 2006 15 603 613 10.1007/s00423-006-0111-z 17031696

[B3] Howard G Wagenknecht LE Cai J Cooper L Kraut MA Toole JF Cigarette smoking and other risk factors for silent cerebral infarction in the general population Stroke 1998 15 913 917 10.1161/01.STR.29.5.913 9596234

[B4] Mannami T Iso H Baba S Sasaki S Okada K Konishi M Tsugane S Cigarette smoking and risk of stroke and its subtypes among middle-aged Japanese men and women: the JPHC study cohort I Stroke 2004 15 1248 1253 10.1161/01.STR.0000128794.30660.e8 15118170

[B5] Miller GJ Bauer KA Cooper JA Rosenberg RD Activation of the coagulant pathway in cigarette smokers Thromb Haemost 1998 15 549 553 9531038

[B6] Mast H Thompson JL Lin IF Hofmeister C Hartmann A Marx P Mohr JP Sacco RL Cigarette smoking as a determinant of high-grade carotid artery stenosis in hispanic, black, and white patients with stroke or transient ischemic attack Stroke 1998 15 908 912 10.1161/01.STR.29.5.908 9596233

[B7] Chalouhi N Ali MS Starke RM Jabbour PM Tjoumakaris SI Gonzalez LF Rosenwasser RH Koch WJ Dumont AS Cigarette smoke and inflammation: role in cerebral aneurysm formation and rupture Mediators Inflamm 2012 15 271582 23316103 10.1155/2012/271582 PMC3532877

[B8] Salzer J Hallmans G Nystrom M Stenlund H Wadell G Sundstrom P Smoking as a risk factor for multiple sclerosis Mult Scler 2013 15 1022 1027 10.1177/1352458512470862 23257617

[B9] Hedstrom AK Hillert J Olsson T Alfredsson L Smoking and multiple sclerosis susceptibility Eur J Epidemiol 2013 15 867 874 10.1007/s10654-013-9853-4 24146047 PMC3898140

[B10] Chang RC Ho YS Wong S Gentleman SM Ng HK Neuropathology of cigarette smoking Acta Neuropathol 2013 15 53 69 24240736 10.1007/s00401-013-1210-x

[B11] Piao WH Campagnolo D Dayao C Lukas RJ Wu J Shi FD Nicotine and inflammatory neurological disorders Acta Pharmacol Sin 2009 15 715 722 10.1038/aps.2009.67 19448649 PMC4002379

[B12] Rosenberg GA Neurological diseases in relation to the blood–brain barrier J Cereb Blood Flow Metab 2012 15 1139 1151 10.1038/jcbfm.2011.197 22252235 PMC3390801

[B13] Hossain M Sathe T Fazio V Mazzone P Weksler B Janigro D Rapp E Cucullo L Tobacco smoke: a critical etiological factor for vascular impairment at the blood–brain barrier Brain Res 2009 15 192 205 19539613 10.1016/j.brainres.2009.06.033 PMC2831230

[B14] Hossain M Mazzone P Tierney W Cucullo L *In vitro* assessment of tobacco smoke toxicity at the BBB: do antioxidant supplements have a protective role? BMC Neurosci 2011 15 92 10.1186/1471-2202-12-92 21943155 PMC3196733

[B15] Valavanidis A Vlachogianni T Fiotakis K Tobacco smoke: involvement of reactive oxygen species and stable free radicals in mechanisms of oxidative damage, carcinogenesis and synergistic effects with other respirable particles Int J Environ Res Public Health 2009 15 445 462 10.3390/ijerph6020445 19440393 PMC2672368

[B16] Raij L Demaster EG Jaimes EA Cigarette smoke-induced endothelium dysfunction: role of superoxide anion J Hypertens 2001 15 891 897 10.1097/00004872-200105000-00009 11393672

[B17] Chen HW Chien ML Chaung YH Lii CK Wang TS Extracts from cigarette smoke induce DNA damage and cell adhesion molecule expression through different pathways Chem Biol Interact 2004 15 233 241 10.1016/j.cbi.2004.09.014 15560890

[B18] Valko M Izakovic M Mazur M Rhodes CJ Telser J Role of oxygen radicals in DNA damage and cancer incidence Mol Cell Biochem 2004 15 37 56 15646026 10.1023/b:mcbi.0000049134.69131.89

[B19] Pryor WA Stone K Zang LY Bermudez E Fractionation of aqueous cigarette tar extracts: fractions that contain the tar radical cause DNA damage Chem Res Toxicol 1998 15 441 448 10.1021/tx970159y 9585474

[B20] Pryor WA Stone K Oxidants in cigarette smoke. Radicals, hydrogen peroxide, peroxynitrate, and peroxynitrite Ann N Y Acad Sci 1993 15 12 27 10.1111/j.1749-6632.1993.tb39148.x 8512242

[B21] Sobczak A Golka D Szoltysek-Boldys I The effects of tobacco smoke on plasma alpha- and gamma-tocopherol levels in passive and active cigarette smokers Toxicol Lett 2004 15 429 437 10.1016/j.toxlet.2004.03.010 15261987

[B22] Dietrich M Block G Norkus EP Hudes M Traber MG Cross CE Packer L Smoking and exposure to environmental tobacco smoke decrease some plasma antioxidants and increase gamma-tocopherol *in vivo* after adjustment for dietary antioxidant intakes Am J Clin Nutr 2003 15 160 166 12499336 10.1093/ajcn/77.1.160

[B23] Willcox JK Ash SL Catignani GL Antioxidants and prevention of chronic disease Crit Rev Food Sci Nutr 2004 15 275 295 10.1080/10408690490468489 15462130

[B24] Masubuchi T Koyama S Sato E Takamizawa A Kubo K Sekiguchi M Nagai S Izumi T Smoke extract stimulates lung epithelial cells to release neutrophil and monocyte chemotactic activity Am J Pathol 1998 15 1903 1912 10.1016/S0002-9440(10)65704-5 9846980 PMC1866325

[B25] Weksler B Romero IA Couraud PO The hCMEC/D3 cell line as a model of the human blood brain barrier Fluids Barriers CNS 2013 15 16 10.1186/2045-8118-10-16 23531482 PMC3623852

[B26] Weksler BB Subileau EA Perriere N Charneau P Holloway K Leveque M Tricoire-Leignel H Nicotra A Bourdoulous S Turowski P Male DK Roux F Greenwood J Romero IA Couraud PO Blood–brain barrier-specific properties of a human adult brain endothelial cell line FASEB J 2005 15 1872 1874 16141364 10.1096/fj.04-3458fje

[B27] Henningfield JE Stapleton JM Benowitz NL Grayson RF London ED Higher levels of nicotine in arterial than in venous blood after cigarette smoking Drug Alcohol Depend 1993 15 23 29 10.1016/0376-8716(93)90030-T 8370337

[B28] Khanna A Guo M Mehra M Royal W III Inflammation and oxidative stress induced by cigarette smoke in Lewis rat brains J Neuroimmunol 2013 15 69 75 10.1016/j.jneuroim.2012.09.006 23031832 PMC3534934

[B29] Abbruscato TJ Lopez SP Roder K Paulson JR Regulation of blood–brain barrier Na, K,2Cl-cotransporter through phosphorylation during *in vitro* stroke conditions and nicotine exposure J Pharmacol Exp Ther 2004 15 459 468 10.1124/jpet.104.066274 15051802

[B30] Hoffmann D Brunnemann KD Prokopczyk B Djordjevic MV Tobacco-specific N-nitrosamines and areca-derived N-nitrosamines: chemistry, biochemistry, carcinogenicity, and relevance to humans J Toxicol Environ Health 1994 15 1 52 10.1080/15287399409531825 8277523

[B31] Fischer S Spiegelhalder B Preussmann R Preformed tobacco-specific nitrosamines in tobacco–role of nitrate and influence of tobacco type Carcinogenesis 1989 15 1511 1517 10.1093/carcin/10.8.1511 2752525

[B32] Djordjevic VB Free radicals in cell biology Int Rev Cytol 2004 15 57 89 15380666 10.1016/S0074-7696(04)37002-6

[B33] Rao R Oxidative stress-induced disruption of epithelial and endothelial tight junctions Front Biosci 2008 15 7210 7226 18508729 10.2741/3223 PMC6261932

[B34] Kong Q Lin CL Oxidative damage to RNA: mechanisms, consequences, and diseases Cell Mol Life Sci 2010 15 1817 1829 10.1007/s00018-010-0277-y 20148281 PMC3010397

[B35] Jain A Flora SJ Dose related effects of nicotine on oxidative injury in young, adult and old rats J Environ Biol 2012 15 233 238 23033686

[B36] Zhou X Sheng Y Yang R Kong X Nicotine promotes cardiomyocyte apoptosis via oxidative stress and altered apoptosis-related gene expression Cardiology 2010 15 243 250 10.1159/000301278 20339300

[B37] Paulson JR Yang T Selvaraj PK Mdzinarishvili A Van der Schyf CJ Klein J Bickel U Abbruscato TJ Nicotine exacerbates brain edema during *in vitro* and *in vivo* focal ischemic conditions J Pharmacol Exp Ther 2010 15 371 379 10.1124/jpet.109.157776 19889792 PMC2812118

[B38] Catanzaro DF Zhou Y Chen R Yu F Catanzaro SE De Lorenzo MS Subbaramaiah K Zhou XK Pratico D Dannenberg AJ Weksler BB Potentially reduced exposure cigarettes accelerate atherosclerosis: evidence for the role of nicotine Cardiovasc Toxicol 2007 15 192 201 10.1007/s12012-007-0027-z 17901562

[B39] Yamaguchi Y Nasu F Harada A Kunitomo M Oxidants in the gas phase of cigarette smoke pass through the lung alveolar wall and raise systemic oxidative stress J Pharmacol Sci 2007 15 275 282 10.1254/jphs.FP0061055 17332694

[B40] McQuaid S Cunnea P McMahon J Fitzgerald U The effects of blood–brain barrier disruption on glial cell function in multiple sclerosis Biochem Soc Trans 2009 15 329 331 10.1042/BST0370329 19143657

[B41] Weiss N Miller F Cazaubon S Couraud PO The blood–brain barrier in brain homeostasis and neurological diseases Biochim Biophys Acta 2009 15 842 857 10.1016/j.bbamem.2008.10.022 19061857

[B42] Deane R Zlokovic BV Role of the blood–brain barrier in the pathogenesis of alzheimer’s disease Curr Alzheimer Res 2007 15 191 197 10.2174/156720507780362245 17430246

[B43] Freeman LR Keller JN Oxidative stress and cerebral endothelial cells: regulation of the blood–brain-barrier and antioxidant based interventions Biochim Biophys Acta 1822 15 822 829 10.1016/j.bbadis.2011.12.009 PMC3412391 22206999

[B44] Coisne C Engelhardt B Tight junctions in brain barriers during central nervous system inflammation Antioxid Redox Signal 2011 15 1285 1303 10.1089/ars.2011.3929 21338320

[B45] Abbott NJ Patabendige AA Dolman DE Yusof SR Begley DJ Structure and function of the blood–brain barrier Neurobiol Dis 2010 15 13 25 10.1016/j.nbd.2009.07.030 19664713

[B46] Liu WY Wang ZB Zhang LC Wei X Li L Tight junction in blood–brain barrier: an overview of structure, regulation, and regulator substances CNS Neurosci Ther 2012 15 609 615 10.1111/j.1755-5949.2012.00340.x 22686334 PMC6493516

[B47] Gavard J Gutkind JS VE-cadherin and claudin-5: it takes two to tango Nat Cell Biol 2008 15 883 885 10.1038/ncb0808-883 18670447 PMC2666287

[B48] Lei Y Stamer WD Wu J Sun X Oxidative stress impact on barrier function of porcine angular aqueous plexus cell monolayers Invest Ophthalmol Vis Sci 2013 15 4827 4835 10.1167/iovs.12-11435 23761078 PMC4594900

[B49] Ley K Leukocytes talking to VE-cadherin Blood 2013 15 2300 2301 10.1182/blood-2013-08-519918 24092927 PMC3790502

[B50] Rajagopalan S Meng XP Ramasamy S Harrison DG Galis ZS Reactive oxygen species produced by macrophage-derived foam cells regulate the activity of vascular matrix metalloproteinases *in vitro*. Implications for atherosclerotic plaque stability J Clin Invest 1996 15 2572 2579 10.1172/JCI119076 8958220 PMC507715

[B51] Ali MA Schulz R Activation of MMP-2 as a key event in oxidative stress injury to the heart Front Biosci (Landmark Ed) 2009 15 699 716 19273096 10.2741/3274

[B52] Shao B Bayraktutan U Hyperglycaemia promotes cerebral barrier dysfunction through activation of protein kinase C-beta Diabetes Obes Metab 2013 15 993 999 10.1111/dom.12120 23617822

[B53] Santaguida S Janigro D Hossain M Oby E Rapp E Cucullo L Side by side comparison between dynamic versus static models of blood–brain barrier *in vitro*: a permeability study Brain Res 2006 15 1 13 10.1016/j.brainres.2006.06.027 16857178

